# Impact and predictors of outcome of COVID-19 in pulmonary hypertension patients

**DOI:** 10.1186/s43168-022-00158-2

**Published:** 2022-11-05

**Authors:** Y. M. A. Soliman, R. I. M. Elkorashy, Ahmed Abdel Aziz, Asmaa Abdelnaby, Sally Magdy

**Affiliations:** 1 Department of Chest Diseases, Faculty of Medicine, Cairo University, Cairo, 11562 Egypt; 2 National heart Institute, Giza, Egypt; 3 Public Health, Faculty of Medicine, Cairo University, Cairo, 11562 Egypt

**Keywords:** Pulmonary hypertension, COVID-19, Impact, Predictors

## Abstract

**Background:**

The pandemic had a significant impact on those with underlying chronic health conditions being at risk of developing a more severe disease with rapid progression, significant complications, and with increased risk of mortality.

This was also expected in the pulmonary vascular community owing to the vulnerable nature of this population, who are characterized by an increase in the pulmonary vascular resistance leading to right heart failure.

This study is aiming to identify the incidence of COVID-19 infection among pulmonary hypertension patients receiving specific therapy as well as the predictors of the COVID-19 disease severity and outcome in those patients.

**Results:**

Data analysis of 197 PAH and CTEPH patients, showed that the incidence of SARS-CoV-2 infection is 10.66% (*n* = 21). Seven patients (33.3%) required hospitalization. Mortality rate is 14.3% (3/21).

Severity of COVID19 disease in those patients has statistically significant moderate to strong correlation with higher values of d-dimer (*r* = 0.821, *P* = 0.000), ferritin (*r* = 0.718, *p* = 0.000), CRP (*r* = 0.613, *p* = 0.04), acute renal failure (*r* = 0.557, *p* = 0.009), and hypoxemia (*r* = 0.825, *p* = 0.000).

Mortality from COVID-19 show moderate to strong statistically significant correlations with acute renal failure (*r* = 0.795, *p* = 0.000), hypoxemia (*r* = 0.645, *p* = 0.002), higher values of ferritin (*r* = 0.689, *p* = 0.001) and d-dimer (*r* = 0.603, *P* = 0.004).

**Conclusions:**

COVID-19 in PAH and CTEPH patients is challenging, higher COVID-19 infection rate is present in those patients and is associated with increased disease severity and higher mortality.

## Introduction and rationale

From the time of emergence of COVID-19 in December 2019, [1] there has been unique challenges facing the healthcare system when dealing with patients with underlying health conditions infected by severe acute respiratory syndrome coronavirus 2 (SARS-CoV-2) as they are at increased risk for poor outcome [2].

The pandemic had a significant impact on those with underlying chronic health conditions being at risk of developing a more severe disease with rapid progression, significant complications, and with increased risk of mortality [3].

This was also expected in the pulmonary vascular community owing to the vulnerable nature of this population, who are characterized by an increase in the pulmonary vascular resistance leading to right heart failure [4].

Putting into consideration the lung injury inflicted via the COVID-19 disease [5] highlights the vulnerability of those patients more than others, as they are probably a main contributor to both morbidity and mortality related to COVID-19 disease.

However, data collected early in the pandemic from pulmonary arterial hypertension (PAH) centers worldwide observed paucity in reported PAH patients infected with COVID-19, and a perceived low risk for severe COVID-19 in PAH patients with an unexpectedly favorable clinical outcome. Multiple clarifications were proposed, some elucidated that the disease itself might be protective against COVID-19, this is suggested by autopsy findings of SARS-CoV-2 infecting endothelial cells with associated vascular injury, thrombosis, and the reduced angiotensin converting enzyme 2 (ACE2) expression that plays a role in reducing the cytokine storm and decreasing the viral entry in PAH patients, others attributed it to PAH-specific medications that showed beneficial effects on COVID-19 pneumonia along with the anticoagulation used in chronic thromboembolic pulmonary hypertension (CTEPH) that reduced the prothrombotic mishaps of the virus [6–9].

Up to date, the available data regarding COVID-19 in pulmonary hypertension is limited, this lack of formal data available regarding COVID-19 infection in pulmonary hypertension (PH) patients, lead to limited scientific-based evidence to guide pulmonary hypertension providers to implement an efficient management approach, as well as to predict the disease course, hence improving survival [10].

This study is aiming to detect the incidence of COVID-19 infection among PH patients receiving specific therapy as well as the prognosticators of the COVID-19 disease severity and outcome in those patients.

## Methodology

A retrospective cohort study of 21 PAH and CTEPH patients infected with COVID-19 seeking medical advice at the Pulmonary Hypertension Centre, Pulmonology Department, Kasr Al-Ainy Hospital from March 2020 to October 2021.

Patients infected with SARS-COV 2 proved positive via real-time polymerase chain reaction (RT-PCR) with or without radiological evidence of COVID-19 pneumonia were included in the study.

Demographic data, presenting symptoms, comorbidities, oxygen saturation, laboratory data, imaging results, medical treatments, hospitalization requirement, disease outcome (death or cure), and risk assessment of pulmonary hypertension during the last scheduled visit are collected from the medical records.

The research ethics committee, Cairo University, Egypt (N-3-2022) has reviewed and approved this study.

The World Health Organization (WHO) classifies COVID-19 severity into mild COVID-19-infected patients showing no clinical signs of pneumonia or hypoxemia, moderate COVID-19 patients showing clinical signs of pneumonia but no hypoxemia, severe COVID-19-infected patients showing clinical signs of severe pneumonia with respiratory rate more than 30 breaths/min, or severe respiratory distress; or hypoxemia with oxygen saturation less than 90% on room air and Critical COVID-19: patient with acute respiratory distress syndrome (ARDS) criteria, septic shock, or requires life-sustaining therapies as mechanical ventilation (invasive or non-invasive) or vasopressor therapy [11].

Pulmonary hypertension patients show an increase in the mean pulmonary artery pressure equal or more than 25 mmHg at rest when measured by the right heart catheter [12].

Assessing the risk in PH patients to estimate 1-year mortality using the prognostic determinants showing that patients with low risk have less than 5% 1-year mortality whereas patients with intermediate risk have from 5 to 10% 1-year mortality and patients with high risk have more than 10% 1-year mortality [12].

Data is collected in Excel sheet, tabulated, and statistically analyzed via SPSS version 21, qualitative data is presented by number and percentage, quantitative data is presented by mean and standard deviation.

Odds ratio is calculated, spearman correlation analysis is done, and chi-square test of significance is done for qualitative data.

Level of significance is set at *p* equal to or below 0.05.

## Results

A total of 197 PAH and CTEPH patients registered in the records of Pulmonary Hypertension Centre, Pulmonology Department, Kasr Al-Ainy Hospital, Cairo University, during the period of March 2020 to October 2021, that yielded 21 confirmed cases of COVID-19, with a percentage of 10.66%. Their mean age was 43.5 ± 11.07 (23-62). Other demographic data, characteristics, clinical presentations and lab findings of the studied patients are summarized in Tables 1 and 2.

**Table 1:** Characteristics and clinical presentation of COVID-19-infected PH cases

Variable	Frequency (21)	%
Gender		
Male	6	28.6
Female	15	71.4
Comorbidities	7	33.3
Chronic liver disease	3	14.3
Diabetes mellitus	1	4.8
Hypothyroidism	1	4.8
Cardiac	1	4.8
Hypertension	1	4.8
Complication Acute renal failure	2	9.5
Pulmonary hypertension diagnosis		
Idiopathic PAH	6	28.6
Schistosomiasis-PH	4	19.0
CTEPH	4	19.0
CTD-PH	3	14.3
Porto-PH	2	9.5
Drug-induced PH	1	4.8
CHD (ES)	1	4.8
PPH-specific treatment regimen		
Dual therapy	14	66.7
Triple therapy	3	14.3
CCB	2	9.5
No	2	9.5
Immunosuppressive treatment	7	33.3
One drug (prednisolone)	4	19.2
Two drugs (prednisolone with another drug)	2	9.5
Three drugs (prednisolone with other drugs)	1	4.8
Anticoagulants		
Yes	4	19.0
No	17	81.0
**Symptoms**		
Fever	12	57.1
Dyspnea	12	57.1
Dry cough	13	61.9
Fatigue	4	19.0
Headache	3	14.3
Chest pain	1	4.8
Hypoxia	6	28.6
Hospitalization	7	33.3
**Severity**		
Mild	8	38.1
Moderate	7	33.3
Severe	2	9.5
Critical	4	19.0
**Outcome**		
Cure	18	85.7
Death	3	14.3

**Table 2:** Laboratory findings of COVID-19-infected PH cases

Lab findings:	Mean ± standard deviation	Range
TL	5.92 ± 2.75	2.2–12
Lymphocytes	1.63 ± 0.59	0.86–2.9
Neutrophil	3.85 ± 2.54	0.6–8.5
Platelets	201.91 ± 58.35	93–351
CRP	42.54 ± 52.14	1–167
Ferritin	213.29 ± 294.66	6–1246
D dimer	0.59 ± 0.37	0.12–1.4

There is no statistically significant relation between risk assessment in the last scheduled visit and the severity and outcome of COVID-19 as shown in Table 3.

**Table 3:** Association between the risk stratification for mortality in PH patients and COVID-19 severity and outcome

Risk assessment	Low risk	Intermediate risk	High risk	*P* value for chi square
Severity:				
Mild	2 (50.0%)	4 (40.0%)	2 (28.6%)	0.408
Moderate	1 (25.0%)	5 (50.0%)	1 (14.3%)	
Severe	0 (0.0%)	1 (10.0%)	1 (14.3%)	
Critical	1 (25.0%)	0 (0.0%)	3 (42.8%)	
Outcome				
Cure	3 (75.0%)	10 (100.0%)	5 (71.4%)	0.259
Death	1 (25.0%)	0 (0.0%)	2 (28.6%)	

There is no statistically significant relation between risk assessment in the last scheduled visit and the severityand outcome of COVID-19.

The presence of fever, high serum ferritin, and d dimer levels had moderate positive significant correlation with the risk assessment of PH in the last scheduled visit as shown in Table 4.

**Table 4:** Correlation between risk stratification of PH in the last scheduled visit and COVID severity, outcome, fever, serum ferritin, and D dimer levels

Risk assessment correlations with:	*P* value for correlation	Pearson *r*
COVID severity	0.210	0.285
Outcome	0.636	0.110
Fever	0.043	0.446
Ferritin	0.050	0.432
D-dimer	0.010	0.548

Presence of fever, high serum ferritin, and d dimer levels had moderate positive significant correlation with risk assessment

The severity of COVID-19 disease in PH cases has statistically significant moderate to strong correlation with higher values of d-dimer, ferritin, and CRP, as well as hypoxemia and acute renal failure as shown in Table 5.

**Table 5:** Correlation between outcome and severity with other factors

Factors		Severity	Mortality
Ferritin	Pearson correlation *r*	.718	.689
	Sig. (2-tailed)	**.000**	**.001**
	*N*	21	21
D dimer	Pearson correlation *r*	.821	.603
	Sig. (2-tailed)	**.000**	**.004**
	*N*	21	21
CRP	Pearson correlation *r*	.613	.279
	Sig. (2-tailed)	**.004**	.233
	*N*	20	20
Acute renal failure	Pearson correlation *r*	.557	.795
	Sig. (2-tailed)	**.009**	**.000**
	*N*	21	21
Hypoxemia	Pearson correlation *r*	.825	.645
	Sig. (2-tailed)	**.000**	**.002**
	*N*	21	21

The mortality of COVID-19 disease in our study population is 14.3%.

Mortality in COVID-19 PH cases has moderate to strong statistically significant correlations with hypoxemia, acute renal failure, higher values of ferritin, and d-dimer as shown in Table 5.

Mortality among COVID-19 PH cases has statistically significant odds ratio with fever, absence of fatigability, hypoxemia, and hospitalization as well as patients who did not receive prednisolone as shown in Table 6.

**Table 6:** Odds ratio for mortality among COVID-19 PH cases

Variable	Death (***n*** = 3)		Cure (***n*** = 18)		Odds ratio	95% CI
Fever	3	100.0	9	50.0	2.000	1.260–3.174
Absence of fatigue	0	0.0	4	22.2	1.286	1.004–1.646
Hypoxemia	3	100.0	3	16.7	6.000	2.136–16.875
Hospitalization	3	100.0	4	22.2	4.500	1.896–10.680
Not receiving prednisolone	3	100.0	11	61.1	1.636	1.132–2.366

## Discussion

The incidence of COVID-19 infection among our study population is 10.66%. (106.6 case per 1000 patients) which when compared to the incidence of COVID-19 infection in the general Egyptian population during the same period (3.3 per 1000) confirms a higher rate of COVID-19 infection among PAH and CTEPH patients [13].

This might be partially explained by realizing that PAH and CTEPH patients are more in contact with the health care system and are medically educated enough to seek help upon recognizing any new symptom, so the probability of them being tested at a higher rate as compared to the general population is present.

Our finding refuted prior studies that reported a relatively limited or similar incidence of COVID-19 infection in comparison to the general population [10, 14].

These studies were conducted in the early phases of the pandemic when both medical knowledge and widespread COVID-19 testing were lacking as well as home isolation and the disrupted follow-up to the patients, all contributing to the underestimation of cases [15].

The authors also noted that morbidity and mortality are discreetly worse in our study population with case fatality rate of 14.3% and hospitalization rate of 33.3% denoting unfavorable disease outcome.

These findings echo the latest reports published via Belge et al. and Lee et al., both reported high COVID-related mortality of 19% and 12% respectively in PAH and CTEPH patients along with higher rates of hospitalization of 70% and 30% respectively [10, 14].

Also, a retrospective study on PAH patients suffered from COVID-19 reported an unusually high mortality rate of 36.36% and hospitalization rate of 81.81% [16].

Comparing the mortality of COVID-19 disease in our study population (14.3%) from March 2020 to October 2021 to those reported for the Egyptian population at large (5.5%) during the same period, reinforces the allegation that PH is a risk factor of mortality in COVID-19 patients with a relative risk of 2.6 [13].

Our results contradict the earlier reports that suggested an attenuated disease course and favorable outcomes, assuming that specific pulmonary arterial hypertension therapy, low ACE2 levels, and anticoagulants provide protection from the pathological changes occurring from the disease [6–9].

These data are based on early information when the COVID-19 was still evolving, so thorough analysis to avoid misleading the pulmonary hypertension community should be done to prevent putting those patients in a greater risk.

Mortality in PH patients with COVID-19 infection is attributed to the observed pathophysiological impacts of the virus on different body organs.

Acute hypoxemic respiratory failure as a sequel to COVID-19 pneumonia in PH patients is a well-recognized outcome that in our experience so far, is associated with a more severe disease and increased mortality.

These findings echo the latest reports that management of COVID-19-infected PAH patients associated with hypoxemia is difficult [17] and that hypoxemia is associated with poor clinical outcomes [18, 19].

Petrilli et al. stated that impaired oxygen status upon hospital admission despite supplemental oxygen is present among the critically ill patients and is coupled with increased mortality [20].

Moreover, hypoxemia might overload the already compromised right ventricle in a patient with decreased cardiorespiratory reserve predisposing to right heart failure [16].

Bearing in mind the risk of developing ARDS in the setting of pulmonary vascular disease as suggested via prior studies [21, 22], posits hypoxemia as a potential predictor of a COVID-19 pneumonia in those group of patients and its existence might be fatal.

Even though the pathogenesis of COVID-19-induced acute kidney injury is still not precisely clear, yet several underlying mechanisms including direct viral injury, ischemia-reperfusion insults, unwarranted cytokine release, thrombotic events, and drug-induced renal injuries have been recognized [23].

Several prior studies have already identified renal failure as a risk factor associated with increased mortality in patients with sepsis and septic shock irrespective of the etiological cause [24, 25].

Our finding that acute renal failure escalates disease severity and its existence among critically ill COVID-19 patients is associated with increased mortality is consistent with other report [26].

Prior studies established that advanced age [1, 27] and male gender [28] are more prone to adverse outcome. Our participants are younger, 43.5 years old at mean age, and mostly females (71.4%); this might justify our findings that despite the higher incidence of the disease in this study population yet more than two thirds of the cases have mild and moderate COVID-19 disease.

In this study, only 19% of our patients presented with critical illness, this percentage is higher when compared to the multicenter retrospective study, performed on 2724 COVID-19 patients, of whom 423 (15.52%) were critically ill, denoting that PH is a risk factor contributing to COVID-19 disease severity [29].

Higher level of D-dimer is a pivotal predictor of disease severity and mortality indicating that the severe inflammation triggered in the critically ill COVID-19 patients [30] results in extensive coagulopathy [31] thus raising the D-dimer level via widespread thrombin generation and fibrinolysis [32].

Our analysis demonstrates a significant increase in the D-dimer levels in the severe and critically ill COVID-19 patients and that disease mortality is significantly associated with higher D-dimer levels.

These findings are in line with Tian et al and Zhou et al. found, both implied that elevated D-dimer level is associated with higher COVID-19 mortality [33, 34].

Also, Zhang et al. concluded that a fourfold increase in D-dimer level on hospital admission is a predictor of disease mortality in COVID-19 patients [35].

Recognizing the biomarkers that mirrors the level of inflammation, ferritin was first in line.

Our data suggests that higher ferritin levels are encountered in the severe and critically ill patients with higher mortality risk.

These results come in line with the meta-analysis conducted by Cheng et al. that revealed that elevated ferritin level is coupled with unfavorable outcome [36].

Similarly, Para et al. states that higher ferritin level is associated with the COVID-19 disease progression [37], advocating that inflammation generates higher ferritin levels that in turn promotes a cascade of events enhancing additional tissue damage [38].

Likewise, it is observed in this study that the CRP level directly correlates with disease severity. Our results come in agreement with Malik et al., showing that the increase in the CRP level is coupled with a more severe disease [39]. Although elevated CRP level reflects the body’s inflammatory state indicating tissue injury, yet it is non-specific and may be ascribed to other causes as secondary bacterial infection [40].

### Study limitations

Our study is limited by its retrospective nature and limited sample size so predicting factors affecting severity and outcome is not possible due to our limited number of observations.

## Conclusions

COVID-19 in PAH and CTEPH patients is challenging, higher COVID-19 infection rate is present in those patients and is associated with increased disease severity and higher mortality.

## Data Availability

All data generated or analyzed during this study are included in this published article.

## Abbreviations

ACE2: Angiotensin-converting enzyme 2
ARDS: Acute respiratory distress syndrome
COVID-19: Coronavirus disease 2019
CRP: C-reactive protein
CTEPH: Chronic thromboembolic pulmonary hypertension
PAH: Pulmonary arterial hypertension
PH: Pulmonary hypertension
RT-PCR: Real-time polymerase chain reaction
SARS-CoV-2: Severe acute respiratory syndrome coronavirus 2
WHO: World Health Organization
